# Machine learning clustering of psychological response trajectories across the first and second waves of the COVID-19 pandemic

**DOI:** 10.3389/fpsyt.2026.1726108

**Published:** 2026-06-26

**Authors:** Fardous Hasan, Farzana Haque, Roberto Ariel Abeldaño Zuñiga, Nourhan M. Aly, Moréniké Oluwátóyìn Foláyan

**Affiliations:** 1 Department of Information Science and Media Studies, University of Bergen, Bergen, Norway; 2 Department of Global Public Health and Primary Care, University of Bergen, Bergen, Norway; 3 Department of Clinical Dentistry, University of Bergen, Bergen, Norway; 4 Haraldsplass Diakonale Sykehus, Bergen, Norway; 5 COVID-19 mental health and wellness (MEHEWE) Study Group, Obafemi Awolowo University, Ile-Ife, Nigeria; 6 Department of Nursing Science, University of Eastern Finland, Kuopio, Finland; 7 Department of Pediatric Dentistry and Dental Public Health, Faculty of Dentistry, Alexandria University, Alexandria, Egypt; 8 Department of Child Dental Health, Obafemi Awolowo University, Ile-Ife, Osun State, Nigeria

**Keywords:** coping, COVID-19, longitudinal study, machine learning, mental health, psychological clusters

## Abstract

**Background:**

The COVID-19 pandemic disrupted daily life globally, leading to increased psychological distress. While many studies have documented mental health trends, few have tracked how person-centered psychological clusters emerge and evolve across pandemic waves.

**Methods:**

This longitudinal study assessed 338 adults (mean age, 38.3 years; 55% female) from 53 countries at two pandemic waves (2020 and 2021). Post-traumatic stress symptoms (PTSS; 15-item scale, α = 0.93), coping strategies (11 binary items), and pandemic stress (15 binary items) were measured using validated instruments; composite scores were constructed by summing item responses. Machine learning clustering (K-means and Gaussian mixture models) evaluated solutions *k* = 2–6 using the silhouette coefficient, Davies–Bouldin index, Calinski–Harabasz score, BIC/AIC, and the gap statistic. The majority of the criteria supported a three-cluster solution. Cross-wave cluster alignment was achieved using the Hungarian algorithm. Person-centered transitions, stability rates, and demographic associations were examined.

**Results:**

Three statistically defined clusters emerged at both waves: a low-distress, high-coping cluster (labeled “resilient”; Wave 1: 35.5%; Wave 2: 29.00%); a low-coping cluster (40.04%; 49.11%); and a high-distress cluster (25.06%; 21.89%). Overall, 55.3% (95% CI [50.0%, 60.5%]) remained in the same cluster across waves. Among those initially in the resilient cluster, 55.8% transitioned to higher-distress clusters by Wave 2. The low-coping cluster showed the greatest stability (68.9%), and 50.6% of those initially in the high-distress cluster transitioned to lower-distress clusters. Net flows shifted toward the low-coping cluster (31 participants). Country income level was associated with cluster membership at both waves (*p* < 0.001) but did not predict individual transitions. Older age, higher education, and employment were associated with the resilient cluster, whereas younger age and unemployment were associated with high distress.

**Conclusion:**

Psychological responses to prolonged crises are dynamic and heterogeneous. Coping capacity and socioeconomic factors are associated with cluster membership, but causal inferences cannot be drawn due to the observational two-wave design and convenience sampling. These findings suggest that interventions could benefit from strengthening coping skills while also addressing structural factors such as education and employment, though further research with more frequent assessments and experimental designs is needed.

## Introduction

The COVID-19 pandemic, declared a global health emergency by the World Health Organization in March 2020, caused widespread disruptions to daily life through lockdowns, travel restrictions, and social distancing measures. Although these measures were essential for controlling viral transmission, they also increased psychological distress across populations worldwide (1, 2). Individuals experienced prolonged social isolation, economic hardship, and persistent health-related fears (3, 4). Consequently, high rates of mental health issues were observed in the general population (5, 6), along with elevated levels of anxiety, post-traumatic stress symptoms (PTSS), and overall psychological distress during and after the pandemic (7, 8). These patterns revealed how deeply the pandemic affected mental wellbeing, exposed gaps in existing mental health support systems, and underscored the need to understand how people’s mental health changed over time (9, 10).

Most research on the mental health effects of the COVID-19 pandemic has relied on cross-sectional studies, which capture psychological states at a single time point and cannot adequately track change over time. Although large-scale studies have documented overall increases in depression and anxiety, these average trends obscure substantial individual differences (11). Some individuals remained psychologically stable, others experienced persistent distress, while still others recovered over time or developed symptoms later (12). These diverse responses highlight the need for person-centered approaches that identify distinct subgroups based on individual psychological patterns rather than relying on population averages. Moreover, many existing studies use group-level comparisons or statistical models that assume homogeneity across populations, potentially overlooking meaningful psychological variation (13).

Although several recent person-centered longitudinal studies examined changes in coping and wellbeing during COVID-19 (14, 15), these investigations used latent cluster methods and typically examined symptom trajectories or coping patterns separately, without integrating both domains within the same statistical cluster. Theoretically, PTSS, coping strategies, and pandemic-related stress are closely related aspects of psychological adaptation and are unlikely to function independently. Higher levels of distress may be linked to reduced coping capacity, while effective coping may help limit the impact of stress. Examining these dimensions together may therefore help identify distinct patterns of psychological responses that might not be captured when each domain is considered separately (16, 17). By combining PTSS (as an indicator of distress), coping strategies (as a modifiable process), and pandemic stress (as a contextual stressor), the present study tests whether observed groupings are primarily symptom-driven versus coping-driven, and whether these domains change together or diverge over time. This integrated approach aims to offer a more complete picture of psychological adaptation during a prolonged crisis than examining any single domain in isolation (14, 15). Specifically, including coping alongside symptoms allows us to examine whether individuals with similar distress levels differ in their coping resources, a distinction with potential implications for intervention targeting.

Unsupervised machine learning (ML) clustering offers a powerful person-centered approach for examining psychological responses over time by identifying distinct subgroups based on complex multidimensional clusters (18, 19). Unlike traditional variable-centered methods, clustering techniques uncover naturally occurring patterns without predefined assumptions (20). Methods such as K-Means and Gaussian mixture models (GMM) are well-suited for psychological data, as they can reveal meaningful patterns that may not correspond to traditional diagnostic categories (21, 22). These approaches also allow researchers to examine how subgroup structures emerge or change over time (16).

Although clustering methods have successfully identified patterns of resilience and mental health outcomes following natural disasters, their application during the COVID-19 pandemic has been relatively limited (23, 24). Some studies have used these techniques to group individuals based on trauma responses (16, 24), but few have focused specifically on psychological changes related to pandemic experiences. Furthermore, few studies have employed comparable methodologies and measurement tools to examine psychological clusters across two time periods. These gaps limit our ability to determine whether the pandemic led to the emergence of new psychological clusters or merely exacerbated pre-existing mental health conditions.

To address these gaps, this study used unsupervised ML to identify distinct psychological clusters based on PTSS, coping strategies, and pandemic-related stress, and to track person-centered transitions between these clusters from the first (2020) to the second (2021) wave of the COVID-19 pandemic. This is exploratory, person-centered analysis describes patterns of psychological response and transition. Additionally, we examined how demographic factors (age, gender, education, and work status) and country income level were associated with cluster membership and longitudinal trajectory class membership.

The research questions for this study are as follows: (1) What distinct psychological clusters emerge based on PTSS, coping strategies, and pandemic-related stress, and how do person-centered transitions between these clusters occur from the first to the second pandemic wave? (2) How are demographic factors and country income level associated with cluster membership and trajectory class membership?

## Methods

### Study design

This study employed a two-wave longitudinal cohort design using data derived from the Mental Health and Health Experiences during COVID-19 (MEHEWE) global survey. The original MEHEWE study consisted of repeated cross-sectional online surveys conducted during the COVID-19 pandemic in 2020 and 2021 across multiple countries, as shown in **Supplementary Figure 1**. For the present analysis, a longitudinal subsample was constructed by identifying individuals who participated in both survey waves. This subsample, referred to as dataset 3 in **Supplementary File 1**, includes matched responses from the same participants collected at the two time points, allowing for within-individual analysis of changes in psychological outcomes over time. The first wave of data collection took place between 1 June and 31 December 2020, and the second wave was conducted between 1 January and 31 December 2021. The use of a longitudinal design enabled the examination of person-centered psychological trajectories across two distinct phases of the pandemic.

### Study participants

Eligibility was restricted to adults capable of completing an online survey. During the first survey wave, participants were invited to provide their email addresses if they were willing to be contacted for follow-up in subsequent phases of the study. Those who consented were re-contacted and invited to participate in the second wave of data collection. A total of 338 participants completed the survey at both time points and were included in the longitudinal cohort. The final analytical sample included participants from 53 countries, reflecting a geographically diverse population.

### Sampling procedure

The original MEHEWE survey employed a convenience sampling strategy using online recruitment methods during the COVID-19 pandemic. Participants were recruited through digital platforms, including social media, WhatsApp groups, and professional and academic email networks facilitated by 49 international collaborators (25). The study achieved global reach, with participants from 53 countries spanning multiple continents, including Nigeria, Ghana, South Africa, the United States, Egypt, India, Pakistan, Mexico, the United Kingdom, Zimbabwe, Canada, and Jordan. Given that participants were drawn from many countries, each with relatively small sample sizes, the analyses were not designed to examine country-level differences. Instead, country income level was used as a contextual variable to capture broader structural variations across settings. Accordingly, clustering analyses were conducted at the individual level, and the identified clusters reflect person-centered psychological patterns rather than national-level differences.

### Data collection

Data were collected using the SurveyMonkey^®^ platform, with a validated questionnaire (26) administered online due to movement restrictions (2). In the original validation study, the PTSS scale demonstrated a strong content validity index (CVI = 0.83), internal consistency, Cronbach’s α = 0.93, and test–retest reliability (intra-class correlation coefficient = 0.89) (26). The first questionnaire administration took place between June 2020 and December 2020, and the second between October 2021 and January 2022. Participants had the option to review and edit their responses before final submission. Given the ongoing COVID-19 restrictions at the time, a web-based survey method was both practical and appropriate.

### Data preprocessing

To prepare the data for clustering, three composite psychological variables—PTSS, coping strategy, and pandemic stress—were constructed for both the first and second waves using identical item sets, response formats, and scoring procedures. Composite scores were derived by summing item-level responses (binary for coping and pandemic stress; five-point Likert for PTSS). Details of each measure are provided below.

1. PTSS symptoms measurement: PTSS symptoms were assessed using 15 items capturing psychological and physiological responses to past stressful experiences. Items assessed the following: (1) disturbing dreams of a stressful experience; (2) disturbing memories, thoughts, or images of a stressful experience; (3) flashbacks or suddenly feeling as if a stressful experience were happening again; (4) feeling upset when reminded of a stressful experience; (5) avoiding activities because they remind you of a stressful experience; (6) physical reactions when reminded of a stressful experience; (7) avoiding thinking about a stressful experience; (8) loss of interest in previously enjoyed activities; (9) emotional numbness or inability to feel loving feelings; (10) feeling that your future will be cut short; (11) difficulty concentrating; (12) irritability or angry outbursts; (13) trouble falling or staying asleep; (14) trouble remembering important parts of a stressful experience; and (15) feeling distant from others. Each item was rated on a five-point Likert scale ranging from “Not at all” (1) to “Extremely” (5). Item responses were summed to create a composite PTSS score with a theoretical range of 15–75. Observed score ranges were 15–71 in the first wave and 15–75 in the second wave.
2. Coping strategy assessment: Coping strategies were measured using 11 binary (Yes/No) self-reported items, each reflecting a specific behavior participants may have used to support their mental health during the COVID-19 pandemic. Behaviors included the following: (1) talking to friends or family on the phone; (2) talking to friends or family via video chat; (3) talking to friends or family in person; (4) exercising at home; (5) exercising outdoors; (6) gardening; (7) meditation; (8) creative activities; (9) learning a new skill; (10) taking breaks from pandemic-related news; and (11) spending time with pets. Responses were summed to create a composite coping score with a theoretical range of 0–11, which was identical across both waves. Observed score ranges were 0–11 in both waves.
3. Pandemic stress assessment: Pandemic stress was measured using a composite index based on 15 binary (Yes/No) items, reflecting the presence or absence of emotional, psychological, and practical challenges experienced during the COVID-19 pandemic. These included the following: (1) anxiety, (2) depression, (3) loneliness, (4) anger, (5) frustration, (6) grief or feelings of loss, (7) changes in sleep patterns, (8) reduced physical activity, (9) fear of contracting COVID-19, (10) fear of transmitting COVID-19, (11) worrying about others, (12) perceived stigma from others, (13) insufficient emotional or social support, (14) confusion about COVID-19, and (15) confusion about where to obtain reliable COVID-19 information. Item responses were summed to create a pandemic-related stress score with a theoretical range of 0–15. Observed score ranges were 0–14 in the first wave and 0–15 in the second wave.

Identical item sets and scoring procedures were applied across both waves for all three measures. Differences in observed minimum and maximum values across waves reflect empirical variation in response distributions, not changes in item content, scale structure, or scoring methods. Binary response formats were used for coping strategies and pandemic stress to support cross-cultural comparability and reduce response burden during a global emergency (26).

Before composite score construction, the factorability and internal consistency of each construct were evaluated within Wave 1 using exploratory factor analysis (EFA), tetrachoric correlations for binary items (coping and pandemic stress), and Pearson correlations for Likert items (PTSS). Sampling adequacy was confirmed by the Kaiser–Meyer–Olkin (KMO) index (> 0.80), and Bartlett’s test of sphericity (*p* < 0.05). Internal consistency was assessed with Cronbach’s α (≥ 0.70) (**Supplementary Table A1a**). Measurement invariance across waves was tested with multi-group confirmatory factor analysis (MG-CFA; WLSMV estimator). Configural and metric invariance were examined for each construct. Metric invariance held for all three (ΔCFI ≥ -0.01, ΔRMSEA ≤ 0.015) (**Supplementary Table A1b**).

### Demography

Gender identity was recoded into three categories: male and female. Employment status was dichotomized: participants employed full-time, part-time, or self-employed were classified as “Employed”; unemployed, students, retired, and other non-working statuses were classified as “Unemployed/Other”. Education level was categorized into four ordered groups: primary, secondary, university, and postgraduate. Country income level was based on the World Bank income classification, grouping participants into high-income, middle-income (upper- and lower-middle), and low-income categories (27).

### Data standardization

To enable meaningful comparisons across periods, raw scores for PTSS symptoms, coping strategies, and pandemic stress were standardized using *z*-scores. Standardized minimum and maximum values for each variable at each wave are presented in **Supplementary Table 2**.

### Data analyses

The analytical pipeline consisted of three stages: cluster analysis, transition analysis, and demographic subgroup analysis. Cluster analysis was performed using both K-Means clustering and GMM. The optimal number of clusters was determined using multiple complementary criteria to avoid reliance on the elbow method alone. Solutions with *k* = 2 through 6 were evaluated using the silhouette coefficient (maximized), Davies–Bouldin index (minimized), Calinski–Harabasz score (maximized), and gap statistic (based on 10 reference datasets) for both methods. For GMM, model selection was further informed by the Bayesian information criterion (BIC) and Akaike information criterion (AIC) (28). All clustering validity metrics were computed separately for each wave and are reported in **Supplementary Table 3**. Although most validation indices supported a three-cluster solution, the silhouette coefficient at Wave 1 marginally favored a two-cluster solution (0.354 vs. 0.311). Given the small difference in silhouette values and the overall convergence of other criteria (Davies–Bouldin, Calinski–Harabasz, gap statistic, and GMM-based indices) across both waves, the three-cluster solution was retained for interpretability and consistency in cross-wave comparisons. Accordingly, the optimal number of clusters was set as *k* = 3 for both waves, and K-Means outperformed GMM on internal validation indices. To assess the structural stability of the selected three-cluster solution, we performed a non-parametric bootstrap stability test using the adjusted Rand index (ARI). For each wave, we generated 100 bootstrap samples of the same size, re-fitted K-Means (*k* = 3), and compared the resulting partitions to the original clustering using the ARI. The mean ARI and its 95% bootstrap confidence interval are also reported in **Supplementary Table 3**.

Person-centered transitions were tracked by matching participants across waves using unique identifiers. Clustering was performed independently at each wave; thus, numeric cluster labels did not correspond directly across time points. Cross-wave cluster equivalence was therefore established using Euclidean distances between standardized cluster centroids, with optimal one-to-one matching determined via the Hungarian algorithm (**Supplementary Table 4**). Second wave cluster labels were re-aligned to match first wave clusters based on this matching. Transition percentages were then calculated from the aligned labels. Stability was defined as the proportion of participants remaining in the same psychological cluster across waves, with 95% confidence intervals calculated using the Wilson score method.

Associations between country income group and cluster membership were examined at each wave using chi-square tests, with Cramér’s *V* reported as a measure of effect size. Logistic regression analyses examined whether income level predicted individual transitions (deterioration from resilient, improvement from high-distress, and stability in low-coping).

Subgroup analyses examined associations between demographic characteristics (gender, education level, employment status, and age) and cluster membership. Chi-square tests were used for categorical variables, and one-way analysis of variance (ANOVA) was used for age. Effect sizes are reported as Cramér’s *V* for categorical variables and *η*^2^ for ANOVA.

To assess whether Wave 1 coping predicts Wave 2 PTSS at the individual level, a linear regression model was fitted. Wave 2 PTSS was regressed on Wave 1 coping strategy score, Wave 1 PTSS, age, gender, education, and country income status.

To illustrate the clustering results, two types of visualizations were implemented. Radar charts were used to compare patterns of PTSS symptoms, coping strategies, and pandemic stress across the first and second waves of the pandemic. Bar charts were used to present standardized mean *z*-scores for each cluster, highlighting differences in psychological variables between the two time points. A Sankey diagram visually showed the transitions between clusters across the two waves, clearly illustrating how participants moved between different psychological clusters.

All data processing, analysis, and visualization were performed using Python 3.11.7, with the numpy, pandas, scikit-learn, seaborn, and matplotlib libraries primarily used for ML tasks. GitHub repository: https://github.com/Fardous07/COVID-19-Mental-Health-Trajectory-Analysis.

## Results

A total of 338 participants were included with a mean age of 38.6 years (SD ≈ 12.0, range = 18–78). Among the participants, 152 were male (45.0%) and 186 were female (55.0%). Educational attainment was distributed as follows: primary education, 0.3% (*n* = 1); secondary education, 11.2% (*n* = 38); university education, 52.1% (*n* = 176); and postgraduate education, 36.4% (*n* = 123). With respect to country income cluster, 14.5% (*n* = 49) resided in high-income countries, 29.6% (*n* = 100) in middle-income countries, and 55.9% (*n* = 189) in low-income countries.

**Figure 1:**
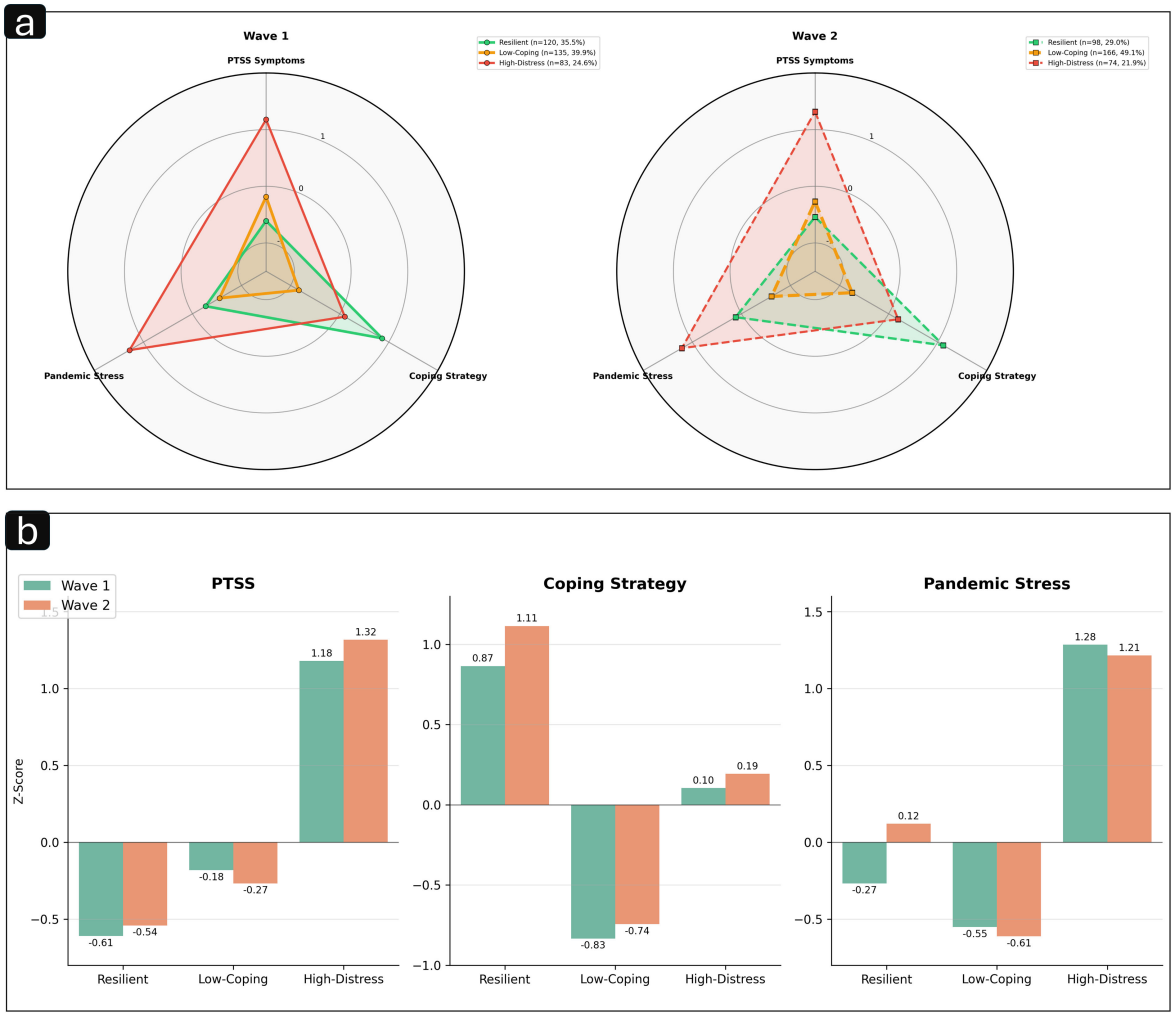
**(A)** Radar plots showing PTSS, coping, and pandemic stress for each cluster at Wave 1 (left, solid lines) and Wave 2 (right, dashed lines); **(B)** bar plot comparison of standardized scores across clusters and waves.

Three empirically derived clusters emerged at each wave based on standardized PTSS symptoms, coping strategies, and pandemic-related stress (**Table 1**; **Figure 1**; **Supplementary Figure 2**). These labels are used descriptively to summarize relative differences in PTSS, coping, and stress scores across the identified groups, and do not represent clinically validated or fixed psychological categories. At Wave 1, the cluster was characterized by the lowest PTSS symptoms, the highest coping scores, and below-average pandemic stress (labeled the resilient cluster, 35.5%). The second cluster showed near-average PTSS symptoms but markedly low coping and reduced pandemic stress (labeled the low-coping cluster, 39.9%). The third cluster demonstrated the highest PTSS symptoms and elevated pandemic stress (labeled the high-distress cluster, 24.6%).

**Table 1:** Mean values of PTSS, coping strategy, and pandemic stress across clusters for the first and second wave of the COVID-19 pandemic (*N* = 338).

Cluster ID	Cluster label	PTSS M ± SD	Coping strategy M ± SD	Pandemic stress M ± SD	*n* (%)
Wave 1					
0	Low-coping	−0.182 ± 0.805	−0.833 ± 0.470	−0.552 ± 0.560	135 (39.9%)
1	High−distress	+1.179 ± 0.845	+0.105 ± 0.911	+1.285 ± 0.833	83 (24.6%)
2	Resilient	−0.610 ± 0.472	+0.865 ± 0.677	−0.268 ± 0.655	120 (35.5%)
Wave 2					
0	High−distress	+1.32 ± 0.91	+0.19 ± 0.81	+1.22 ± 0.96	74 (21.9%)
1	Low-coping	−0.27 ± 0.71	−0.74 ± 0.47	−0.61 ± 0.48	166 (49.1%)
2	Resilient	−0.54 ± 0.51	+1.11 ± 0.61	+0.12 ± 0.77	98 (29.0%)

Following cross-wave alignment using Euclidean distances and the Hungarian algorithm (**Supplementary Table 4**), the same three clusters retained their core structural characteristics but shifted in prevalence. At Wave 2, the resilient clusters (29.0%) again combined low PTSS and high coping, though stress was now near the sample average. The low-coping cluster (49.1%) remained defined by near-average PTSS and low coping. The high-distress cluster (21.9%) continued to show elevated PTSS and high pandemic stress, with coping close to the mean.

**Figure 2:**
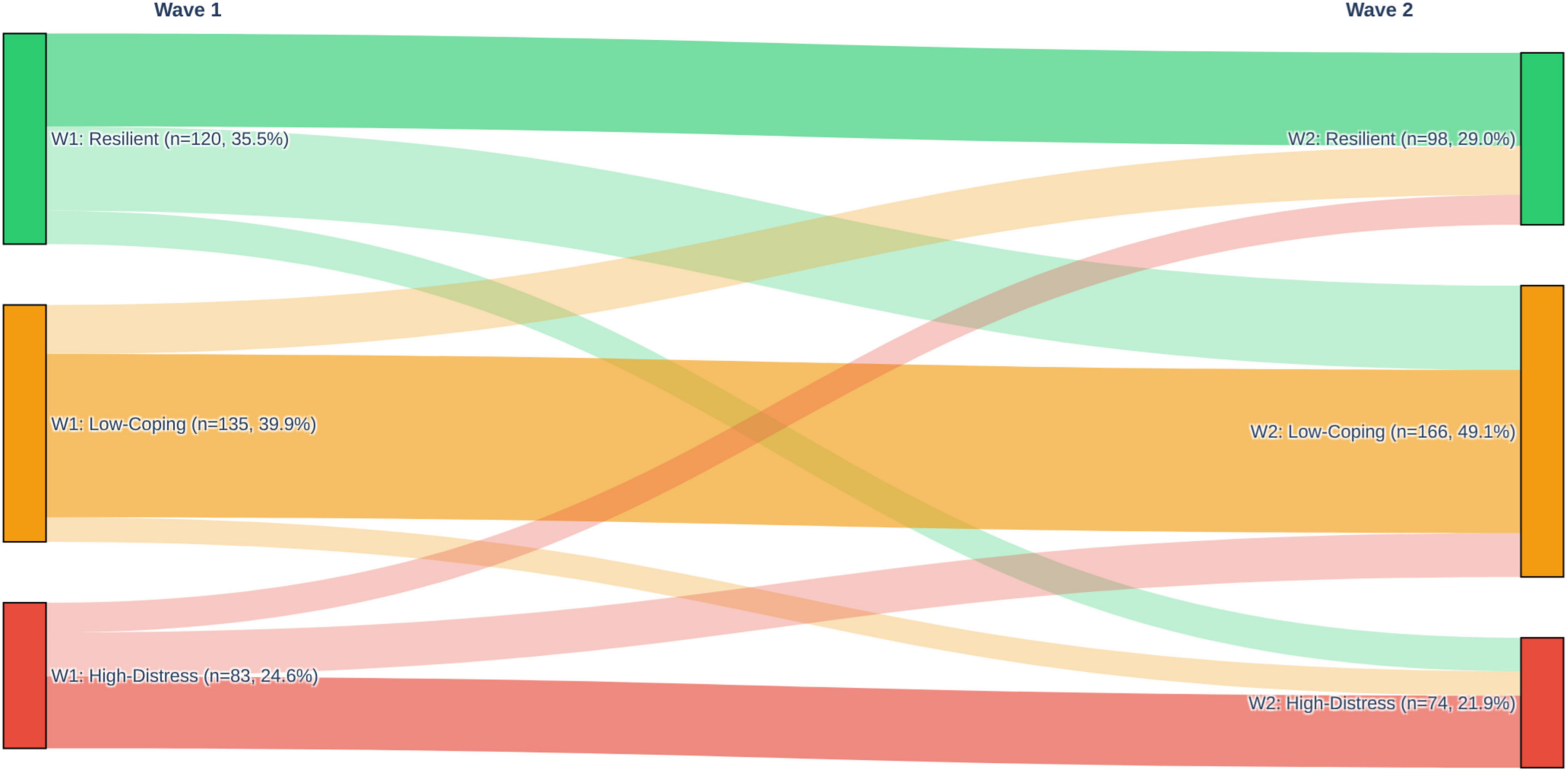
Sankey diagram showing participant flow between clusters from Wave 1 to Wave 2.

Person-centered transitions in psychological clusters from Wave 1 to Wave 2 are presented in **Table 2** and illustrated in **Figure 2**. Of the participants, 55.3% remained in the same cluster across waves, while 44.7% shifted to a different cluster.

**Table 2:** Individual-level transitions between psychological clusters from Wave 1 to Wave 2 (*N* = 338).

Wave 2 Wave 1	Resilient	Low-coping	High-distress	Stability (95% CI)
Resilient	53 (44.2%)	48 (40.0%)	19 (15.8%)	44.2% [35.6%, 53.1%]
Low-coping	28 (20.7%)	93 (68.9%)	14 (10.4%)	68.9% [60.6%, 76.1%]
High-distress	17 (20.5%)	25 (30.1%)	41 (49.4%)	49.4% [38.9%, 59.9%]

The resilient cluster was the least stable. Fewer than half of those initially classified as resilient (44.2%) retained that status at Wave 2. The majority (55.8%) moved into other clusters: 40.0% into the low-coping cluster and 15.8% into the high-distress cluster. By contrast, the low-coping cluster exhibited the highest stability (68.9%). When individuals in this group changed, the shift was far more likely to be toward resilience (20.7%) than toward high distress (10.4%). Among those initially in the high-distress cluster, 49.4% remained in the same cluster at Wave 2. The remaining 50.6% moved to a less symptomatic state: 30.1% transitioned to moderate distress, and 20.5% achieved resilience.

Country income level was significantly associated with cluster membership at both waves (**Table 3**). At Wave 1, the low-coping cluster was more prevalent among low-income participants (44.4%) than among high-income participants (16.3%), while the high-distress cluster was notably more common in the high-income group (46.9%) than in the low-income group (17.5%). The resilient cluster varied less across income levels, though it was somewhat higher among low-income (38.1%) and high-income (36.7%) participants than among middle-income participants (30.0%). At Wave 2, the low-coping cluster dominated the low-income group (58.5%), far exceeding its presence in the high-income group (27.3%). The high-distress cluster again showed greater representation among high-income participants (31.8%) relative to low-income participants (15.4%). The resilient cluster displayed a clear income gradient, with 40.9% of high-income participants classified as resilient compared to 26.2% of low-income participants.

**Table 3:** Country income level distribution across psychological clusters at Wave 1 and Wave 2.

Wave	Income level	Resilient *n* (%)	Low-coping *n* (%)	High-distress *n* (%)	χ² (*p*)	*V*
Wave 1	High income	18 (36.7)	8 (16.3)	23 (46.9)	23.38 (<0.001)	0.186
	Middle income	30 (30.0)	43 (43.0)	27 (27.0)		
	Low income	72 (38.1)	84 (44.4)	33 (17.5)		
Wave 2	High income	18 (40.9)	12 (27.3)	14 (31.8)	20.92 (<0.001)	0.176
	Middle income	29 (29.3)	40 (40.4)	30 (30.3)		
	Low income	51 (26.2)	114 (58.5)	30 (15.4)		

In contrast, country income level did not significantly predict individual-level transitions between clusters (**Supplementary Table 5**). Logistic regression analyses yielded no significant effects for deterioration from the resilient cluster, improvement from the high-distress cluster, or stability in the low-coping cluster.

Gender did not differ significantly across profile clusters at either wave, indicating that profile cluster membership was not differentiated by gender. In contrast, education, work status, and age were consistently associated with cluster membership at both waves (**Table 4**). The resilient cluster had the highest proportion of postgraduate education and the highest employment rates, while the high-distress cluster had the lowest. Age showed a medium-sized effect, with resilient participants being the oldest and high-distress participants the youngest.

**Table 4:** Demographic characteristics by trajectory class.

Characteristic	Stable resilient (*n* = 53)	Deteriorated (*n* = 67)	Stable moderate (*n* = 93)	Improved (*n* = 45)	Stable high distress (*n* = 41)
Age (years)					
Mean ± SD	44.6 ± 12.9	41.3 ± 13.0	37.6 ± 10.8	40.0 ± 11.9	33.3 ± 10.3
95% CI	[41.3, 48.2]	[38.5, 44.5]	[35.3, 39.7]	[36.6, 43.3]	[30.3, 36.4]
*p* < 0.001, *η*^2^ = 0.08					
Gender, *n* (%)					
Female	32 (60.4%)	34 (50.7%)	51 (54.8%)	22 (48.9%)	25 (61.0%)
Male	21 (39.6%)	33 (49.3%)	42 (45.2%)	23 (51.1%)	16 (39.0%)
*p* = 0.67, *V* = 0.09					
Education, *n* (%)					
Primary	0 (0.0%)	0 (0.0%)	1 (1.1%)	0 (0.0%)	0 (0.0%)
Secondary	3 (5.7%)	8 (11.9%)	10 (10.8%)	3 (6.7%)	7 (17.1%)
University	19 (35.8%)	29 (43.3%)	58 (62.4%)	27 (60.0%)	21 (51.2%)
Postgraduate	31 (58.5%)	30 (44.8%)	24 (25.8%)	15 (33.3%)	13 (31.7%)
*p* < 0.001, *V* = 0.28					
Work status, *n* (%)					
Employed	44 (83.0%)	47 (70.1%)	62 (66.7%)	29 (64.4%)	15 (36.6%)
Unemployed	9 (17.0%)	20 (29.9%)	31 (33.3%)	16 (35.6%)	26 (63.4%)
*p* < 0.001, *V* = 0.27					
Country income, *n* (%)					
High income	8 (15.1%)	10 (14.9%)	5 (5.4%)	10 (22.2%)	13 (31.7%)
Middle income	14 (26.4%)	16 (23.9%)	24 (25.8%)	16 (35.6%)	13 (31.7%)
Low income	31 (58.5%)	41 (61.2%)	64 (68.8%)	19 (42.2%)	15 (36.6%)
*p* = 0.003, *V* = 0.20					

**Deteriorated = Resilient → low-coping or high-distress; Improved = low-coping or high-distress → Resilient.

To examine predictors of longitudinal patterns, five trajectory classes were defined (a sixth, mixed-transition group, comprising 11.5% of the sample, was excluded from trajectory analyses): stable resilient (15.7%), deteriorated (19.8%), stable moderate (27.5%), improved (13.3%), and stable high-distress (12.1%). Age differed significantly across trajectory classes (η² = 0.08, medium effect). Stable resilient participants were the oldest and stable high-distress participants were the youngest, with a large effect size between these two extremes (*d* = 0.96). Gender showed no significant differences across trajectory classes. Education differed significantly (*V* = 0.28), with postgraduate education most prevalent in the stable resilient class (58.5%) and least prevalent in the stable high-distress class (31.7%). Work status was the strongest predictor (*V* = 0.27) with employment rates declining progressively from stable resilient (83.0% employed) to stable high-distress (36.6% employed). Country income also differed significantly across trajectory classes (*V* = 0.20, small effect). Notably, the proportion of participants from high-income countries was highest in the stable high-distress class (31.7%) and substantially lower in the stable resilient class (15.1%). Low-income country participants were most concentrated in the stable moderate (68.8%) and deteriorated (61.2%) classes. The mixed-transition group (*n* = 39, 11.5%) included two subgroups: participants who transitioned from high-distress to low-coping (partial improvement; *n* = 25, 7.4%) and those who transitioned from low-coping to high-distress (worsening; *n* = 14, 4.1%).

In **Table 5**, Wave 1 coping strategy did not significantly predict Wave 2 PTSS after controlling for baseline PTSS and demographic variables. Of the included predictors, only baseline PTSS and age were statistically significant. Gender, education level, and country income level were not significantly associated with Wave 2 PTSS. The model explained 40.7% of the variance in Wave 2 PTSS (adjusted *R*² = 0.407). Regression coefficients are presented as unstandardized estimates (*B*) with 95% confidence intervals.

**Table 5:** Longitudinal predictors of PTSS at Wave 2.

Predictor	*B*	95% CI	*p*
Coping strategy (W1)	0.18	[−0.26, 0.61]	0.43
PTSS symptoms (W1)	0.56	[0.47, 0.65]	<0.001
Age	−0.09	[−0.18, −0.002]	0.04
Gender	1.51	[−0.61, 3.63]	0.16
Education	−0.81	[−2.67, 1.05]	0.39
Country income level	−0.12	[−1.08, 0.83]	0.80

## Discussion

This study applied a person-centered ML approach to identify distinct clusters of relative psychological response patterns across two waves of the COVID-19 pandemic. It also tracked how individuals transitioned between these clusters over time. Three consistent clusters emerged: resilient, low-coping, and high-distress. While the structural characteristics of each cluster remained stable across waves, individual-level cluster membership was highly dynamic. These findings show that psychological changes during prolonged crises are not uniform or fixed, as resilience, vulnerability, and recovery can coexist and change within the same population over time.

The study’s strengths include its longitudinal design and a person-centered analytical framework that tracked the same participants across two pandemic waves. This approach allowed the detection of within-subject psychological changes while minimizing cohort effects and enabled the integration of multiple psychological domains. Methodologically, the use of multiple clustering techniques, validation procedures, and bootstrap iterations contributed to the stability and reliability of the identified clusters.

Despite its strengths, this study has several limitations. Participants from multiple countries had varying contexts, but small per-country samples precluded direct country-level analyses, leaving non-significant associations with country income as a proxy. In addition, coping and pandemic stress were assessed with binary items, limiting sensitivity, and the resulting composite scores are simplified approximations, a consequence of the MEHEWE survey’s rapid, early pandemic design using simple binary behaviors rather than validated instruments. Furthermore, the convenience sampling overrepresented educated, digitally connected, and English-proficient individuals, likely underrepresenting marginalized groups. Of over 19,000 Phase 1 participants, only 338 (∼1.8%) provided follow-up data, and because no Wave 1 exists for non-consenters, attrition bias cannot be formally tested; thus, all longitudinal findings apply only to this small, self-selected subgroup and are exploratory, not population estimates. Also, although convenience sampling was the only feasible method for rapid cross-national data during the emergency, prevalence and transition rates describe only this sample. Finally, only two time points and no pre-pandemic baseline preclude causal interpretations and prior mental health status determination. Nevertheless, the current study provides useful insights.

The central finding of this study is that psychological responses during the COVID-19 pandemic were dynamic rather than fixed for many individuals. A substantial proportion of the sample transitioned between clusters across waves, implying that they experienced meaningful psychological change over the inter-wave period. This aligns with previous research demonstrating heterogeneous mental health trajectories following large-scale stressors, including pandemics, disasters, and trauma (11, 16). Notably, resilience did not represent a stable or enduring state for many individuals. Only 44.2% of initially resilient participants remained resilient, while 40.0% transitioned to moderate distress and 15.8% moved to high distress. Conversely, half of those initially experiencing high distress moved to a less distressed cluster (50.6%), with 30.1% moving to moderate distress and 20.5% achieving resilience. This pattern suggests that single-point assessments may overestimate the stability of the low-distress, high-coping cluster across a prolonged crisis (11). Together, these results are consistent with a process-oriented conceptualization of resilience, emphasizing fluctuation and adaptation rather than stable personality traits (29). The present study extends this literature by integrating PTSS symptoms, coping capacity, and pandemic-related stress within a single person-centered framework, offering a more nuanced understanding of how these domains jointly shape psychological adaptation over time.

The low-coping cluster emerged as the largest and most stable group across both waves. Importantly, this cluster was defined not by severe symptom burden but by persistently limited coping capacity. Even when symptom levels declined, poor coping remained a defining feature. This finding reinforces theoretical and empirical work showing that coping processes play a central role in long-term psychological adjustment and may precede changes in symptom severity (30).

This finding highlights that symptom reduction alone does not necessarily reflect increased resilience. Individuals with low coping capacity may remain psychologically vulnerable despite experiencing fewer immediate stressors. This pattern highlights the importance of evaluating coping processes alongside symptom levels, particularly during prolonged periods of stress (17). Coping capacity may serve as a potential indicator of longer-term mental health risk, providing potential critical insight into vulnerability that may not be captured by symptom measures alone (31). Accordingly, longitudinal mental health monitoring should incorporate assessments of coping strategies in addition to symptom severity to more accurately identify individuals at risk for sustained psychological difficulties.

Sociodemographic analyses revealed consistent associations between psychological clusters and age, education, and employment status. Across both waves, resilient clusters were characterized by older age, higher educational attainment, and higher employment rates, whereas high-distress clusters were disproportionately composed of younger and unemployed individuals. These findings echo extensive evidence that socioeconomic resources are associated with lower psychological stress, possibly through increased material security, perceived control, and access to adaptive coping resources (32, 33).

Although country income level was associated with cluster membership at each wave, it did not predict individual-level transitions over time. This pattern suggests that individual socioeconomic positioning, such as education and employment, may exert a stronger influence on psychological trajectories than the broader national income context, consistent with prior work showing that within-country inequalities often outweigh between-country differences in mental health outcomes (32, 34). At the same time, the presence of persistent high distress among participants from high-income countries cautions against assuming that macro-level wealth uniformly protects against psychological vulnerability.

These findings suggest several tentative implications for mental health responses during prolonged crises. These implications should be interpreted cautiously and are intended to generate hypotheses rather than serve as direct evidence of intervention effectiveness. First, the instability of resilience suggests that continuous monitoring may be valuable, though further research is needed to confirm this. Second, the prominence and persistence of the low-coping cluster suggest that coping-focused interventions could be explored in future research, particularly for individuals with low coping capacity, even when symptoms are moderate. Third, the strong associations between psychological resilience and structural factors such as education and employment indicate that effective responses to large-scale crises should integrate psychological support with socioeconomic measures. Policies that promote employment stability, educational access, and economic security may be associated with reduced vulnerability. However, causal inference would require experimental or quasi-experimental designs (35, 36).

## Conclusion

This exploratory study suggests that psychological responses to prolonged crises can be dynamic and heterogeneous, shaped by coping capacity and socioeconomic resources. By documenting both resilience loss and recovery across pandemic waves, the findings illustrate the potential value of person-centered approaches for understanding mental health dynamics over time. The findings also suggest that assessing coping capacity alongside symptoms may provide additional insight, as symptom reduction alone was not sufficient to ensure cluster stability. Interventions during prolonged crises should therefore address both psychological processes and structural factors. These findings suggest that interventions targeting coping skills, as well as policies that improve educational access and employment stability, could be explored in future experimental studies to determine their effectiveness in reducing psychological distress during prolonged crises. Future research should include more frequent assessments and examine how cultural and policy contexts shape psychological adaptation over time. Such multilevel approaches may help reduce long-term psychological consequences, though this requires further investigation.

## Data Availability

The raw data supporting the conclusions of this article will be made available by the authors, without undue reservation.
