# Supplementary figures and images for: Machine learning clustering of psychological response trajectories across the first and second waves of the COVID-19 pandemic
Source: Front Psychiatry. 2026 Jun 26;17:1726108. doi: 10.3389/fpsyt.2026.1726108 (PMC13350411; doi:10.3389/fpsyt.2026.1726108)

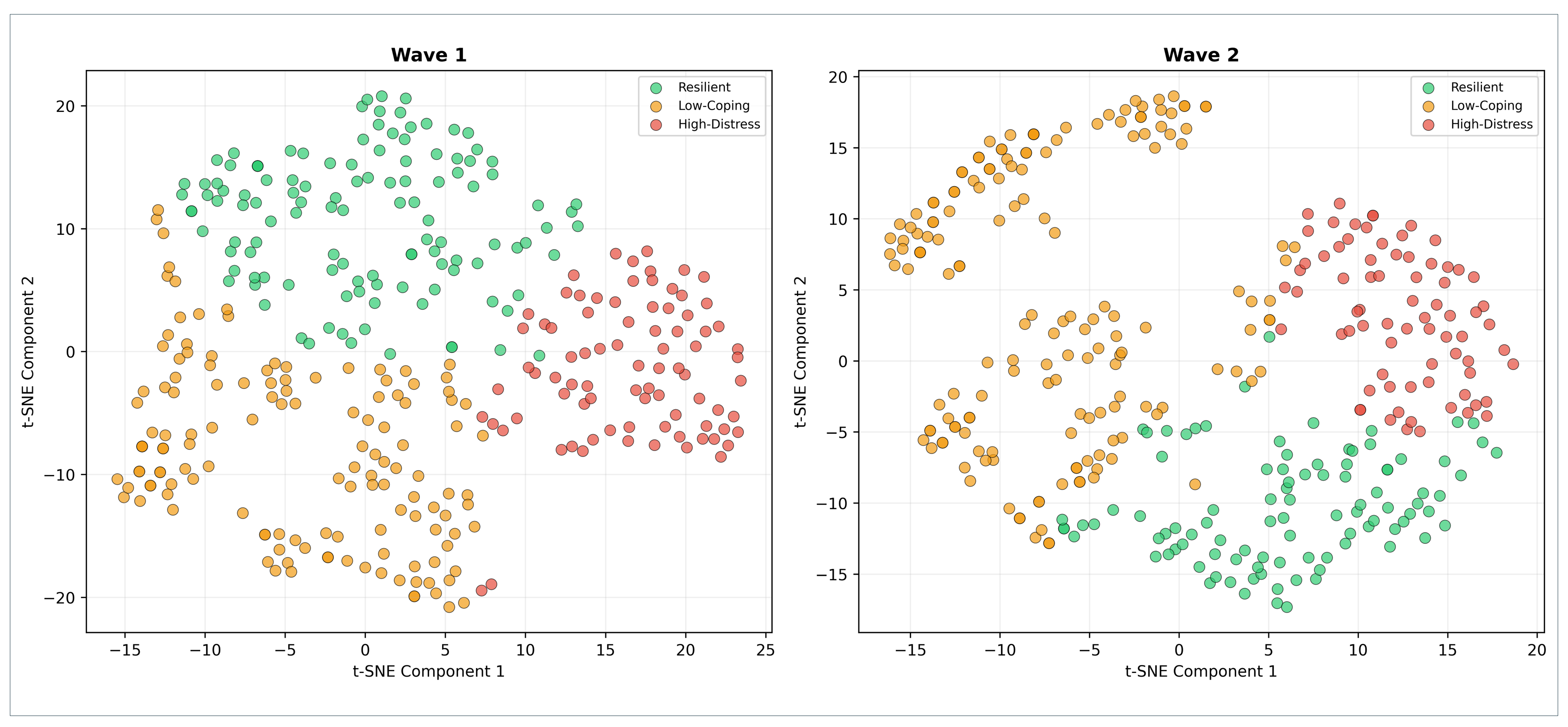

Supplement: Supplementary file 1 [file Image1.png]
