# Supplementary material for: Machine learning clustering of psychological response trajectories across the first and second waves of the COVID-19 pandemic
Source: Front Psychiatry. 2026 Jun 26;17:1726108. doi: 10.3389/fpsyt.2026.1726108 (PMC13350411; doi:10.3389/fpsyt.2026.1726108)
Supplement: Supplementary file 2 [file SupplementaryFile1.docx]

**APPENDIX**

**Supplementary** **Table 1a:** Psychometric properties of the PTSS, coping, and pandemic stress scales.

| **Construct** | **KMO** | **Bartlett’s test (*p*)** | **Cronbach’s α** |
| --- | --- | --- | --- |
| Wave 1 | | | |
| PTSS | 0.943 | <0.001 | 0.942 |
| Coping strategy | 0.740 | <0.001 | 0.671 |
| Pandemic stress | 0.785 | <0.001 | 0.785 |
| Wave 1 | | | |
| PTSS | 0.948 | <0.001 | 0.949 |
| Coping strategy | 0.845 | <0.001 | 0.804 |
| Pandemic stress | 0.850 | <0.001 | 0.838 |

**Supplementary** **Table 1b.** Configural and metric invariance across waves.

| **Construct** | **Configural CFI** | **Configural RMSEA** | **Metric ΔCFI** | **Metric ΔRMSEA** | **LRT *p*‑value** |
| --- | --- | --- | --- | --- | --- |
| PTSS | 0.930 | 0.153 | +0.0214 | -0.0302 | 0.2859 |
| Coping strategy | 0.962 | 0.052 | +0.0081 | -0.0082 | 0.6156 |
| Pandemic stress | 0.936 | 0.059 | +0.0145 | -0.0090 | 0.6981 |

**Supplementary** **Table 2.** Standardized minimum and maximum values for PTSS symptoms, coping strategy, and pandemic stress during the first and second pandemic periods.

| **Period** | **PTSS symptoms**  **(Min → Max)** | **Coping strategy**  **(Min → Max)** | **Pandemic stress**  **(Min → Max)** |
| --- | --- | --- | --- |
| First wave | -1.10 **→** 3.05 | -1.75 **→** 2.76 | -1.23 **→** 3.40 |
| Second wave | -1.21 **→** 3.63 | -1.11 **→** 2.84 | -0.99 **→** 3.61 |

**Supplementary** **Table 3.** Clustering performance metrics for K-Means and GMM across *k* = 2-6.

| **Wave** | **k** | **Method** | **Silhouette** | **Davies Bouldin** | **Calinski Harabasz** | **BIC** | **AIC** | **Gap Statistic (SE)** |
| --- | --- | --- | --- | --- | --- | --- | --- | --- |
| Wave 1 | 2 | K Means | 0.36 | 1.22 | 173.97 | — | — | 0.49 (0.02) |
|  |  | GMM | 0.30 | 1.37 | 153.01 | 2,714.77 | 2,642.13 | — |
|  | 3 | K Means | 0.31 | 1.07 | 187.51 | — | — | 0.59 (0.03) |
|  |  | GMM | 0.19 | 1.28 | 96.21 | 2,685.22 | 2,574.35 | — |
|  | 4 | K Means | 0.30 | 1.13 | 168.10 | — | — | 0.49 (0.03) |
|  |  | GMM | 0.18 | 1.47 | 95.15 | 2,715.18 | 2,566.08 | — |
|  | 5 | K Means | 0.30 | 1.11 | 159.46 | — | — | 0.43 (0.01) |
|  |  | GMM | 0.24 | 1.24 | 124.23 | 2,795.95 | 2,608.62 | — |
|  | 6 | K Means | 0.30 | 1.05 | 159.89 | — | — | 0.42 (0.04) |
|  |  | GMM | 0.14 | 1.38 | 93.88 | 2,746.24 | 2,520.68 | — |
|  | K-Means (*k* = 3): Mean ARI = 0.91, 95% CI = [0.79, 1.00] | | | | | | | |
| Wave 2 | 2 | K Means | 0.35 | 1.24 | 187.27 | — | — | 0.53 (0.03) |
|  |  | GMM | 0.18 | 1.11 | 82.93 | 1,752.59 | 1,679.95 | — |
|  | 3 | K Means | 0.36 | 1.02 | 205.14 | — | — | 0.55 (0.04) |
|  |  | GMM | 0.24 | 1.17 | 130.84 | 1,616.01 | 1,505.14 | — |
|  | 4 | K Means | 0.34 | 1.07 | 193.58 | — | — | 0.55 (0.04) |
|  |  | GMM | 0.25 | 1.23 | 138.76 | 1,637.92 | 1,488.82 | — |
|  | 5 | K Means | 0.32 | 1.09 | 187.35 | — | — | 0.49 (0.04) |
|  |  | GMM | 0.27 | 1.15 | 145.78 | 1,632.23 | 1,444.90 | — |
|  | 6 | K Means | 0.32 | 1.07 | 180.03 | — | — | 0.42 (0.02) |
|  |  | GMM | 0.27 | 1.23 | 136.30 | 1,680.65 | 1,455.09 | — |
|  | K-Means (*k* = 3): Mean ARI = 0.77, 95% CI = [0.60, 0.92] | | | | | | | |

**Supplementary Table 4.** Cross-wave cluster centroid distance matrix and Hungarian algorithm matching.

| **Wave 2**  **Wave 1** | **Cluster 0** | **Cluster 1** | **Cluster 2** | **Matched To** | **Distance** |
| --- | --- | --- | --- | --- | --- |
| Cluster 0: Low-coping (PTSS = -0.182, Coping = -0.833, Stress = -0.552) | 2.53 | **0.14** | 2.09 | Wave 2: Cluster 1 (low-coping) | 0.14 |
| Cluster 1: High‑distress (PTSS = +1.179, Coping = +0.105, Stress = +1.285) | **0.18** | 2.53 | 2.31 | Wave 2: Cluster 0 (high‑distress) | 0.18 |
| Cluster 2: Resilient (PTSS = -0.610, Coping = +0.865, Stress = -0.268) | 2.52 | 1.68 | **0.47** | Wave 2: Cluster 2 (resilient) | 0.47 |

**Supplementary Table 5.** Logistic regression predicting transitions by country income level.

| **Outcome** | **Comparison** | **OR** | **95% CI** | ***p*-value** |
| --- | --- | --- | --- | --- |
| Deterioration from resilience | High vs. low income | 0.95 | [0.33, 2.67] | 0.92 |
| Improvement from high distress | High vs. low income | 0.64 | [0.22, 1.87] | 0.42 |
| Stability in moderate | Low vs. high income | 1.92 | [0.42, 8.75] | 0.40 |

**Eligible for follow-up**

**Participants who provided email contact for future waves (n = 700+)**

**Wave 1 (June-Dec 2020)**

**~19000+ participants**

**(80+ countries, convenience sample)**

**Responded to Wave 2**

**n = 338**

**Final analytic sample (used for all clustering and transition analyses)**

**n = 338**

**Supplementary Figure 1.** Data collection flow chart.


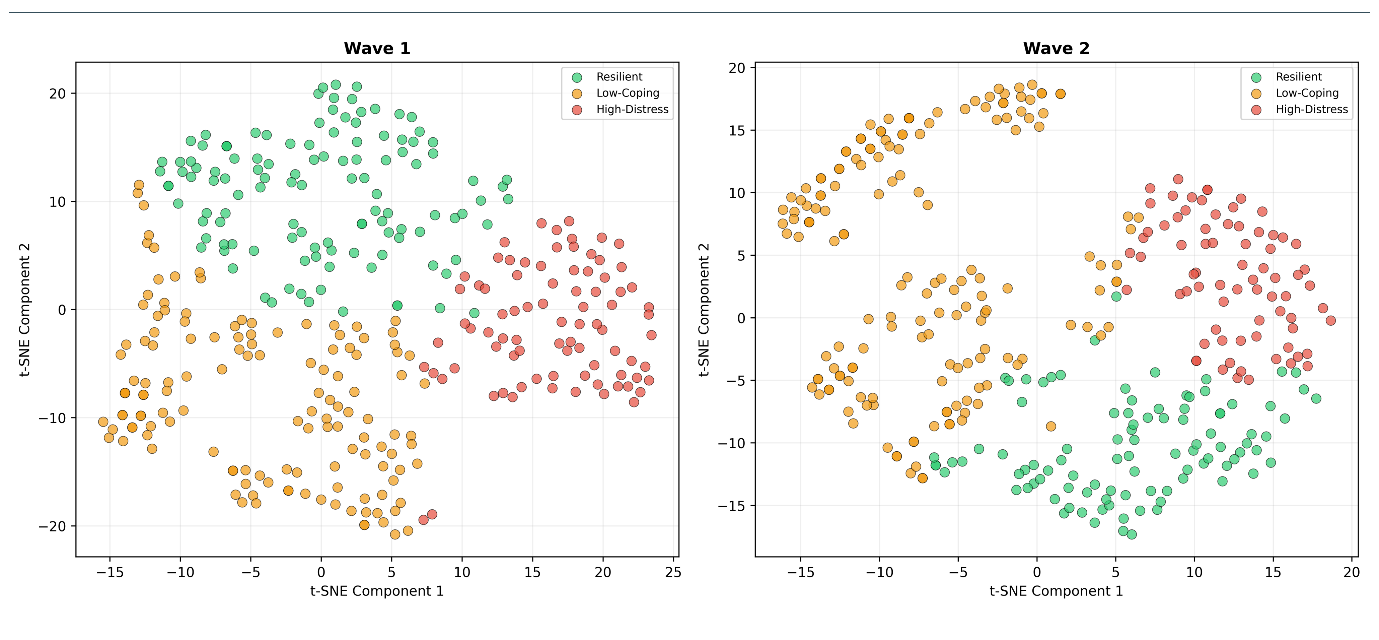
**Supplementary Figure 2.** t‑SNE visualization of the three‑cluster solution derived from K‑means clustering across Wave 1 and Wave 2.
